# Supplementary material for: Improved detection and consistency of RNA-interacting proteomes using DIA SILAC
Source: Nucleic Acids Res. 2024 Jan 9;52(4):e21. doi: 10.1093/nar/gkad1249 (PMC10899761; doi:10.1093/nar/gkad1249)
Supplement: gkad1249_Supplemental_Files [file gkad1249_supplemental_files.zip › Tan et al SI.pdf]

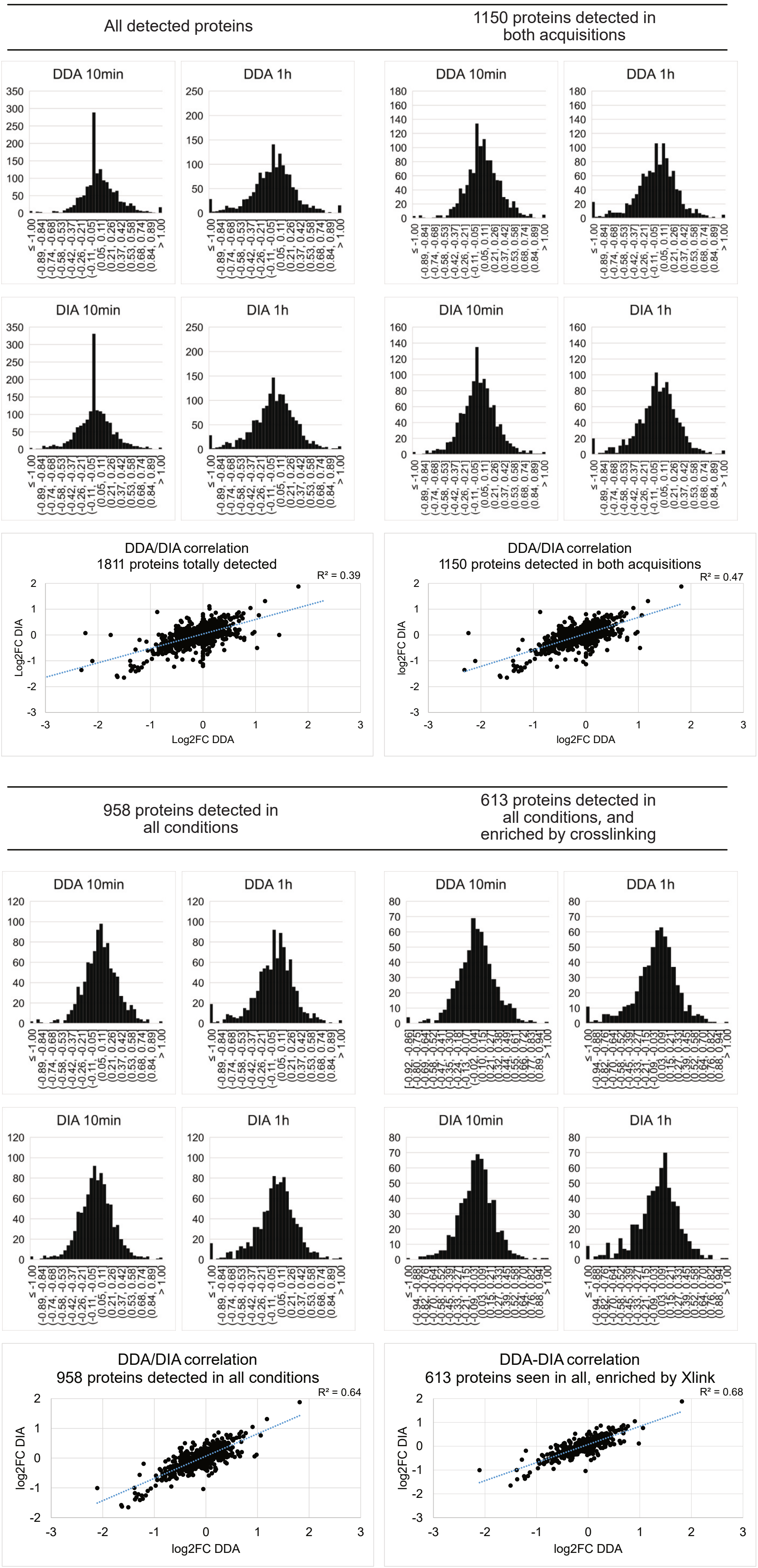

Tan et al Fig. S1

Fig.S1. Distribution of Light:Heavy ratios and correlations of log2FC values between DDA and DIA datasets for 10 min and 1 h samples

Values were calculated from normalized, processed datasets. The DDA dataset was normalized by MaxQuant, DIA by Cyclic Loess. Separate plots are shown for; All detected proteins; Proteins detected in both acquisitions; Proteins detected in all conditions; Proteins detected in all conditions and enriched by UV-crosslinking.

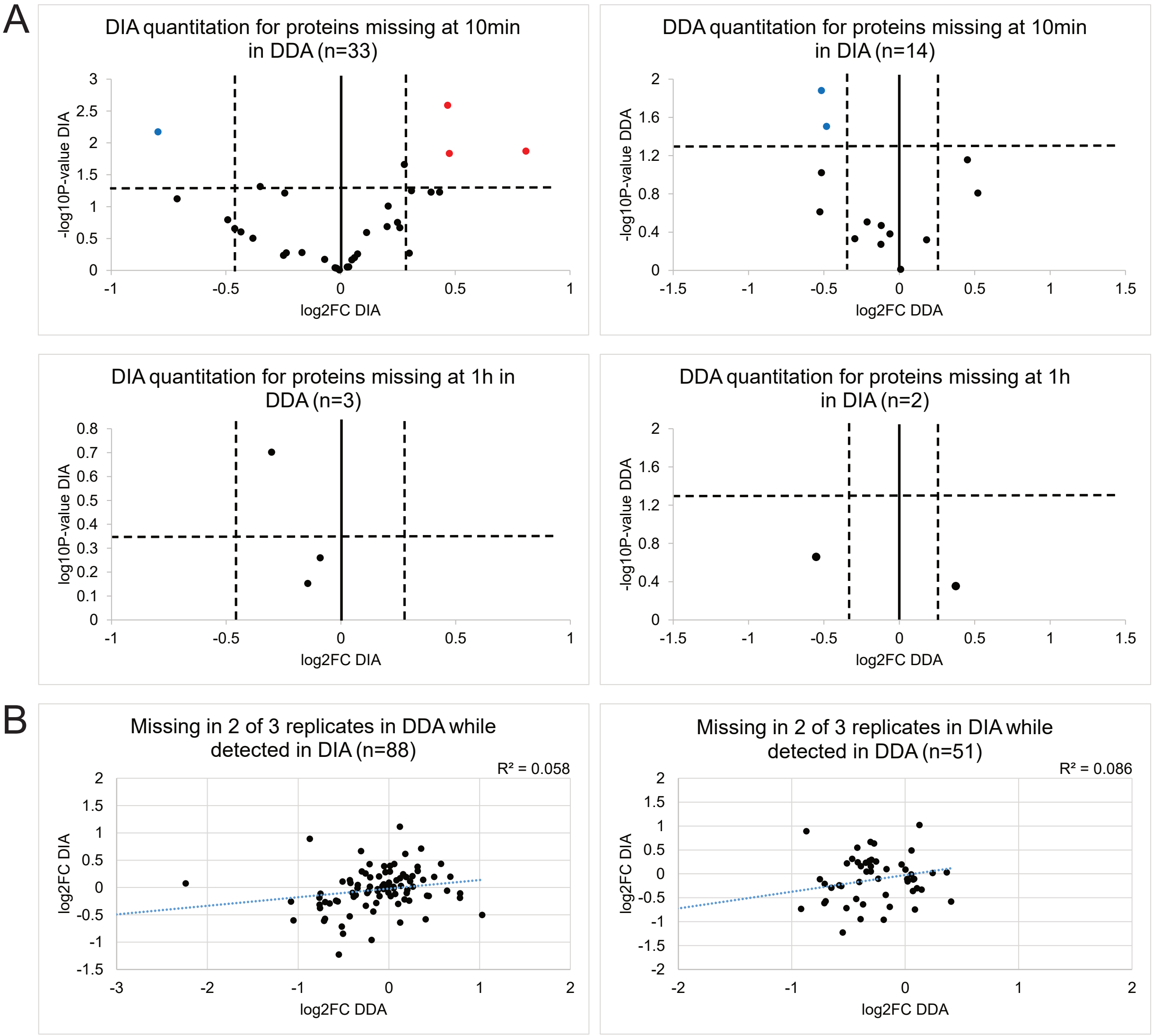

Tan et al Fig. S2

Fig.S2. Proteins totally absent or with 2 missing values at any time point could not be consistently quantified between DDA and DIA datasets. (A) Volcano plot showing DDA or DIA quantitation for proteins totally missing at either 10 min or 1 h in the other acquisition. (B) DDA-DIA correlation for proteins with 2 missing values at any time point while also detected in the other acquisition.

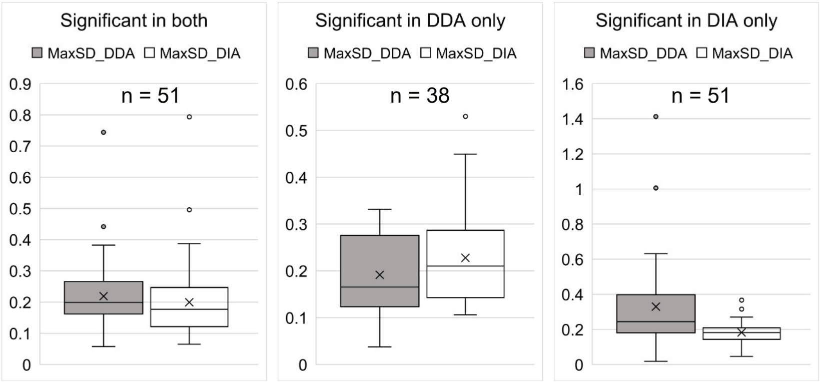

Tan et al Fig. S3

Fig.S3. Inter-replicate variations of proteins significant in both DDA and DIA, or exclusively in each dataset. Variations were represented by the higher standard deviation in Light:Heavy ratios between replicates in 10min or 1h (MaxSD). Center line, median; box limits, upper and lower quartiles; whiskers, 1.5x interquartile range; points, outliers.

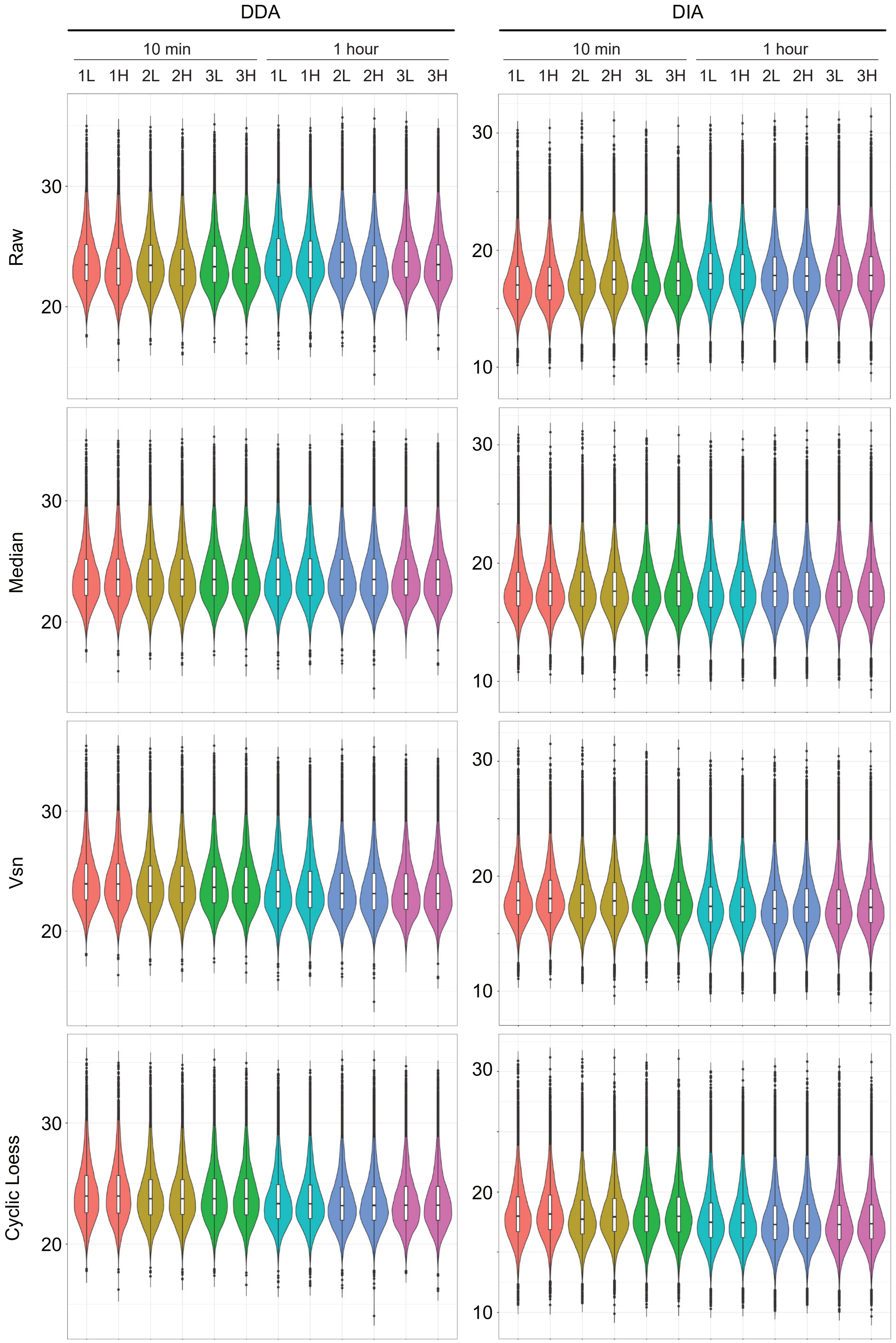

Tan et al Fig. S4

Fig.S4. Effects of different normalization algorithms on distributions of peptide intensities. Raw intensities of individual precursors were normalized using median, Vsn, and Cyclic Loess in NormalyzerDE and plotted as violin and box plots. NA values were excluded. Center line, median; box limits, upper and lower quartiles; whiskers, 1.5x interquartile range; points, outliers.

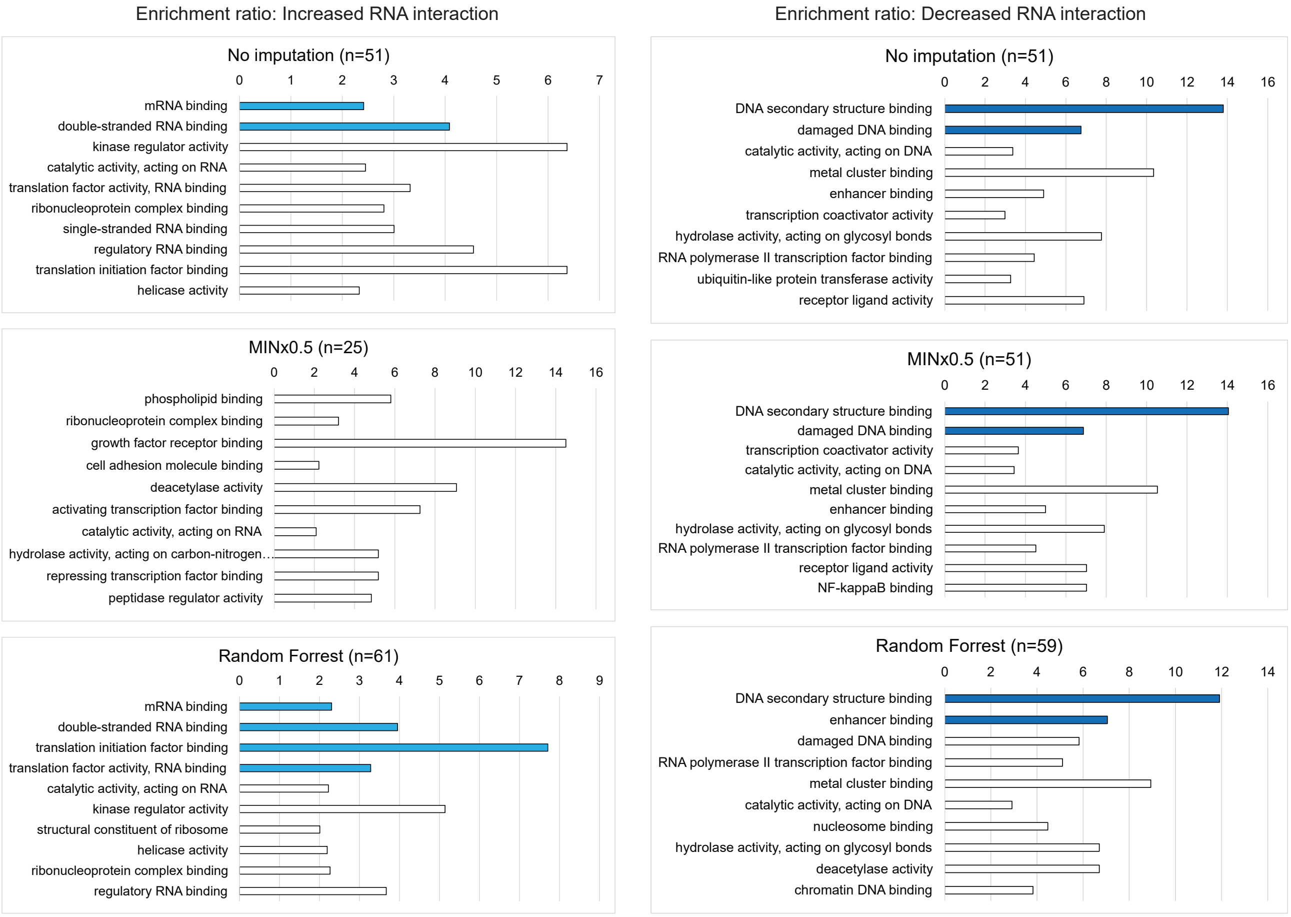

Tan et al Fig. S5

Fig.S5. Top ten enriched GO terms for molecular function in datasets with: No imputation; Imputation by MINx0.5; Imputation by Random Forrest. GO term enrichment was analyzed for proteins with significantly increased (left) or decreased (right) RNA interactions from 10 min and1 h samples in the DIA datasets. These were selected using 2-sided T Test: P value <0.05 and fold-change >90th and <10th percentile. Dark blue bar, FDR<0.05; pale blue bar, FDR between 0.05-0.06; white bar, FDR>0.05. GO terms were sorted by FDR in ascending order, or by enrichment ratio if all FDRs were 1.

Increase RNA Interaction

No imputation	MINx0.5	Random Forrest
ATXN2	**DBNL**	ATXN2
BMS1	DNAJC7	BMS1
CCDC9	**EIF2A**	CCDC9
CPEB4	**MBNL3**	CPEB4
CSTF1	**MRPL22**	CSTF1
DCAF13	**MRPS25**	**DCAF13**
DDX3X	**MRPS35**	DDX3X
DDX54	**NMD3**	DDX54
DDX6		DDX6
		DHX15
**DHX9**		**DHX9**
DKC1		DKC1
		DNAJC7
EIF3A		**EIF3A**
		EIF3B
EIF4A1		EIF4A1
EIF4B		EIF4B
EIF4H		EIF4H
FXR2		FXR2
		IMPDH2
IQGAP1		IQGAP1
KRR1		KRR1
MFAP1		MFAP1
MRPL10		MRPL10
MRPL2		MRPL2
MRPL34		MRPL34
NOB1		NOB1
		NUDT21
PARP12		PARP12
PATL1		PATL1
PEBP1	PEBP1	PEBP1
PES1	**PHAX**	PES1
	**PRCC**	PITPNB
PRPF8	RNF214	**PRPF8**
PRR3	**RPP25L**	PRR3
PRRC2A	**RPP30**	PRRC2A
PRRC2C	SAMD4B	PRRC2C
PUM1	**SIN3A**	PUM1
PUM2	**SMNDC1**	PUM2
R3HDM1	**SNX2**	R3HDM1
R3HDM2	**SUPV3L1**	R3HDM2
RBFOX2	**THUMPD1**	RBFOX2
RBM19	**TRNAU1AP**	RBM19
RBM8A	TUT1	RBM8A
	**YTHDF3**	RNF40
RPA1	ZC3H15	RPA1
RPL17	**ZC3H6**	RPL17
		RPL18A
RPL29		RPL29
RPL35		RPL35
		RPS16
RPS24		RPS24
**SART3**		**SART3**
TRIM25		TRIM25
UTP15		UTP15
WDR75		WDR75
WNK1		WNK1
YTHDC2		YTHDC2
ZC3HAV1		ZC3HAV1
		ZCCHC3
ZNF622		ZNF622

Decrease RNA Interaction

No imputation	MINx0.5	Random Forrest
ATAD5	**APRT**	ATAD5
BAZ1A		
BAZ1B	BAZ1B	BAZ1B
BAZ2A	BAZ2A	BAZ2A
BCOR	BCOR	BCOR
CCDC124	CCDC124	CCDC124
CCDC86	CCDC86	CCDC86
	**CDKN2A**	CDCA2
CHAF1A	CHAF1A	CHAF1A
CHAMP1	CHAMP1	CHAMP1
	**COPZ1**	CNOT4
DRG1	DRG1	DRG1
EDF1	**EDF1**	**EDF1**
FCF1	FCF1	FCF1
G3BP1	G3BP1	G3BP1
GNL3L	GNL3L	GNL3L
GTF3C1	GTF3C1	GTF3C1
		HDAC1
HDLBP	HDLBP	HDLBP
		HERC5
HMGB1	HMGB1	HMGB1
HMGB2	HMGB2	HMGB2
HMGB3	HMGB3	HMGB3
**HNRNPA0**	**HNRNPA0**	**HNRNPA0**
INCENP	INCENP	INCENP
JUN	JUN	JUN
KDM3B	**KDM3B**	KDM3B
		KIF22
LIG3	LIG3	LIG3
LTV1	LTV1	LTV1
MGA	**MGA**	MGA
MKI67	MKI67	MKI67
MPG	MPG	MPG
MTDH	MTDH	MTDH
	NEDD8	MTERF1
		NUSAP1
PDCD4	PDCD4	PDCD4
POLE	POLE	POLE
PSMC3	PQBP1	PSMC3
RAI1	RAI1	RAI1
		RBBP4
RBM42	RBM42	RBM42
RIF1	RIF1	RIF1
RPL11		
RPS10	RPS10	RPS10
**RPS14**	**RPS14**	**RPS14**
**RPS28**	**RPS28**	**RPS28**
RPS3	**RPS3**	RPS3
RPS5	**RPS5**	RPS5
		SECISBP2
		SLC4A1AP
**SND1**	**SND1**	**SND1**
SRP68	SRP68	SRP68
TARDBP	TARDBP	TARDBP
TMPO	**TMPO**	**TMPO**
TRIP12	**UBE2N**	TTF2
UBE2D2		
UHRF1	UHRF1	UHRF1
**UPF1**	**UPF1**	**UPF1**
		USP48
**XPC**	**XPC**	**XPC**
**ZC3H11A**	**ZC3H11A**	**ZC3H11A**

Table S1. List of proteins showing significant changes in the three datasets. Dark grey shaded, consistent between all three; Pale grey shaded, consistent between no imputation and Random Forrest; Bold black font, proteins significant after Benjamini-Hochberg multiple test correction, FDR<0.05.

Significant proteins

Similarity (%)	37.3	54.3	41.2	63.6
	**In silico up**	**Project specific up**	**In silico down**	**Project specific down**
	ATXN2	ATXN2	ATAD5	
	BMS1		BAZ1A	
	CCDC9	CCDC9	BAZ1B	BAZ1B
	CPEB4		BAZ2A	
	CSTF1		BCOR	BCOR
	DCAF13		CCDC124	
	DDX3X	DDX3X	CCDC86	
	DDX54	DDX54	CHAF1A	CHAF1A
	DDX6		CHAMP1	CHAMP1
	**DHX9**	DHX9	DRG1	CHTF18
	DKC1			CKAP4
	EIF3A	EIF3A		CNOT4
	EIF4A1	EIF4A1	EDF1	EDF1
	EIF4B	EIF4B	FCF1	EMD
		EIF4G2	G3BP1	FNDC3A
	EIF4H	**EIF4H**	GNL3L	
		FMR1	GTF3C1	GTF3C1
	FXR2	FXR2	HDLBP	HP1BP3
	IQGAP1		HMGB1	
	KRR1	KRR1	HMGB2	
	MFAP1	LSM6	HMGB3	
	MRPL10	LSM8	**HNRNPA0**	
	MRPL2	METTL16	INCENP	
	MRPL34	MRPS2	JUN	
	NOB1	NOC3L	KDM3B	
	PARP12	NUDT21	LIG3	LIG3
	PATL1	PPAN	LTV1	LTV1
	PEBP1			MECP2
	PES1		MGA	MGA
	PRPF8		MKI67	MKI67
	PRR3	PRR3	MPG	MPG
	PRRC2A		MTDH	MTDH
	PRRC2C	PRRC2C	PDCD4	
	PUM1	PUM1	POLE	POLE
	PUM2	PUM2	PSMC3	RBBP4
	R3HDM1	**R3HDM1**	RAI1	RBM5
	R3HDM2	R3HDM2	RBM42	RBM7
	RBFOX2	RBM34	RIF1	
	RBM19	RBMXL1	RPL11	RPL11
	RBM8A		RPS10	
	RPA1	RPA1	**RPS14**	RPS14
	RPL17	RPL17	**RPS28**	RPS28
	RPL29	RPL3	RPS3	RPS3
	RPL35	RPS2	RPS5	RPS5
	RPS24	SAFB		SBDS
	**SART3**	TRUB1		SLC4A1AP
	TRIM25	UPF3B	**SND1**	**SND1**
	UTP15		SRP68	
	WDR75		TARDBP	
	WNK1		TMPO	TMPO
	YTHDC2		TRIP12	
	ZC3HAV1		UBE2D2	
	ZNF622		UHRF1	UHRF1
			**UPF1**	
			**XPC**	
			**ZC3H11A**	

Table S2. List of proteins showing significant changes in DIA datasets analyzed with *in silico* or project-specific spectral libraries. Level of similarity between the two analyses are indicated above each list. Significance threshold: 2-sided T Test P-value <0.05, FC >90th and <10th percentile. Proteins shown in bold font indicate those deemed significant after Benjamini Hochberg multiple test correction (FDR<0.05).
